# Educational action to monitor children’s growth and development based on the theory of meaningful learning

**DOI:** 10.1590/1980-220X-REEUSP-2023-0200en

**Published:** 2024-01-12

**Authors:** Daniele de Souza Vieira, Paloma Karen Holanda Brito, Iolanda Carlli da Silva Bezerra, Anniely Rodrigues Soares, Luciano Marques dos Santos, Beatriz Rosana Gonçalves de Oliveira Toso, Elenice Maria Cecchetti Vaz, Neusa Collet, Altamira Pereira da Silva Reichert

**Affiliations:** 1 Universidade Federal da Paraíba, João Pessoa, PB, Brazil.; 2 Universidade Estadual de Feira de Santana, Feira de Santana, BA, Brazil.; 3 Universidade Estadual do Oeste do Paraná, Cascavel, PR, Brazil.

**Keywords:** Primary Health Care, Child Development, Education, Nursing, Public Health Surveillance, Atención Primaria de Salud, Desarrollo Infantil, Educación, Enfermería, Vigilancia de la Salud Pública, Atenção Primária à Saúde, Desenvolvimento Infantil, Educação, Enfermagem, Vigilância em Saúde Pública

## Abstract

**Objective:**

Evaluating the effect of an educational program on the knowledge of Primary Health Care nurses regarding the surveillance of growth and development during childcare appointments.

**Method:**

A before-after intervention study with 30 nurses. The nurses’ knowledge and practice assessment were done using a tool that had been developed and validated. The educational activity was carried out, linking child growth and development indicators with public policies for early childhood and nurses’ practices. David Ausubel’s Significant Learning Theory was used as a teaching-learning strategy. The same instrument was reapplied after one month. Descriptive statistics were used in the analysis and the proportion test, Wilcoxon test and Item Response Theory with the Rasch model were applied.

**Results:**

The nurses checked more items right in the instrument after the intervention; there was an increase in the scores of correct answers and a decrease in the item response difficulty index in the knowledge and practice section, post-intervention.

**Conclusion:**

The educational activity had a positive effect, affecting changes in nurses’ knowledge and practice, which enabled childcare consultations to become more qualified.

## RESUMO

**Objetivo:**

Avaliar o efeito de uma ação educativa no conhecimento de enfermeiros da Atenção Primária à Saúde, para a vigilância do crescimento e do desenvolvimento na consulta de puericultura.

**Método:**

Estudo de intervenção do tipo antes-depois realizado com 30 enfermeiros. Avaliou-se o conhecimento e a prática dos enfermeiros mediante instrumento construído e validado. Realizou-se a atividade educativa, articulando indicadores de crescimento e desenvolvimento infantil com políticas públicas da primeira infância e as práticas do enfermeiro. Utilizou-se a Teoria da Aprendizagem Significativa de David Ausubel como estratégia de ensino-aprendizagem. O mesmo instrumento foi reaplicado após um mês. Na análise, utilizou-se estatística descritiva e aplicaram-se os testes de proporção, Wilcoxon e a Teoria da Resposta ao Item com o modelo de *Rasch.*

**Resultados:**

Os enfermeiros acertaram mais itens do instrumento após a intervenção; houve elevação dos escores de acertos e diminuição do índice de dificuldade de resposta ao item na seção do conhecimento e da prática, pós-intervenção.

**Conclusão:**

A atividade educativa teve efeito positivo, repercutindo mudanças do conhecimento e da prática dos enfermeiros, a qual possibilitou que a consulta de puericultura se tornasse mais qualificada.

## RESUMEN

**Objetivo:**

Evaluar el efecto de un programa educativo sobre los conocimientos de las enfermeras de atención primaria acerca del seguimiento del crecimiento y el desarrollo durante las consultas de puericultura.

**Método:**

Estudio de intervención antes-después realizado con 30 enfermeras. Se evaluaron los conocimientos y la práctica de las enfermeras mediante un instrumento elaborado y validado. Se realizó la actividad educativa, articulando los indicadores de crecimiento y desarrollo infantil con las políticas públicas para la primera infancia y las prácticas de las enfermeras. Se utilizó la Teoría del Aprendizaje Significativo de David Ausubel como estrategia de enseñanza-aprendizaje. El mismo instrumento fue reaplicado después de un mes. En el análisis se utilizó estadística descriptiva y se aplicó la prueba de proporciones, la prueba de Wilcoxon y la Teoría de Respuesta al Ítem con el modelo de Rasch.

**Resultados:**

Las enfermeras acertaron más ítems del instrumento después de la intervención; hubo un aumento en las puntuaciones de respuestas correctas y una disminución en el índice de dificultad de respuesta al ítem en la sección de conocimientos y práctica, post-intervención.

**Conclusión:**

La actividad educativa tuvo un efecto positivo, que se tradujo en cambios en los conocimientos y la práctica de las enfermeras, lo que permitió que las consultas de puericultura fueran más cualificadas.

## INTRODUCTION

Care activities directed at early childhood, the period that corresponds to the first six years of a child’s life, has the potential to positively impact child development, offering a greater possibility of motor, cognitive and socio-emotional gains ^(1)^.

Child health care in Brazil has gone through a long historical process of evolution and improvement of public policies, starting with a model centered on disease and curative actions and evolving to a model based on a broader view of health, contemplating, in addition to disease prevention and cure, health promotion and protection. As a result, important milestones were reached, such as the fall in infant mortality rates, the expansion of access to services, greater vaccination coverage and a reduction in malnutrition. In this process, nursing has become an important player in child care, due to its potential to provide comprehensive care and health promotion practices^(2)^.

Regarding child care, Primary Health Care (PHC) is highlighted as a privileged place to provide basic care, where childcare nursing consultations take place, which have the potential to promote health, carrying out Child Development Surveillance (CDS)^(3)^ to monitor growth and development, carrying out at least seven consultations in the first year of life and once a year after the age of two^(4)^.

Nurses play an important role in children’s preventative consultations, caring for the health of infants and their families, and are a key player in monitoring children’s well-being in health services^(5)^. This consultation is carried out systematically and routinely, in which techniques are implemented to monitor the child’s growth and state of health, assess neuropsychomotor development, check and advise on breastfeeding, immunization, among others, with the aim of promoting children’s health^(6)^.

Although its importance for comprehensive care in PHC and the reduction of morbidity and mortality in children has been proven^(7)^, studies show that the care actions carried out by nurses in childcare consultations are compromised, as most of these professionals do not assess all the parameters recommended for monitoring children’s growth and development^(3,8)^.

A United States study analyzing the screening and developmental surveillance of 5,668 children concluded that follow-up rates in CDS remain low, highlighting the need to improve the quality of care in order to provide children with a consultation capable of detecting developmental delays early on, as well as intervening in health problems in a timely and efficient manner^(9)^.

On the other hand, a study carried out in New Zealand found that an educational intervention carried out with nurses on developmental surveillance significantly improved their knowledge and practice with regard to assessing for developmental milestones and signs suggestive of autism^(10)^. An investigation in Iran showed that the educational intervention carried out with nurses significantly improved their knowledge and practice, contributing to a reduction in hospital-acquired infections^(11)^.

Given the evidence of the fragility of nurses’ actions in childcare consultations and the success of educational interventions to overcome these limitations, further training is needed to integrate nursing knowledge and skills in the context of Primary Care^(12)^. In view of the above, this research is based on the hypothesis that an educational intervention based on the Significant Learning Theory (SLT) can change the knowledge and practice of nurses, linking child growth and development indicators with public policies for comprehensive child health care, with a focus on early childhood and actions to protect, recover and promote child health.

David Ausubel’s SLT presents extremely important concepts applied to the teaching-learning process and highlights the importance of awakening the learner to learn from what they already know, providing integration between what is new and what has already been experienced, in order to broaden their knowledge and transform their reality^(13)^.

Given the scarcity of research in Brazil analyzing the effect of an educational intervention with nurses who work in PHC, for the surveillance of child growth and development in childcare consultations, based on SLT, this study contributes to overcoming this gap in scientific knowledge in Brazilian nursing.

For that reason, we asked ourselves: what is the effect of an educational intervention on the knowledge and practice of nurses who work in PHC, regarding child growth and development surveillance actions in childcare appointments? In order to answer this question, the aim was to evaluate the effect of an educational action on the knowledge of nurses working in Primary Health Care regarding the surveillance of growth and development in childcare appointments, based on the Significant Learning Theory.

## METHOD

### Type of Study

This was a quasi-experimental before-after single-group study^(14)^, guiding its stages using an instrument that assesses nurses’ knowledge and practice in surveillance of child growth and development in the Family Health Strategy (FHS), adopting Raymundo’s assumptions^(15)^ for its validation. The Standards for Quality Improvement Reporting Excellence (SQUIRE) guideline was used to guarantee the quality of the research.

### Site

The research site was the Family Health Team (FHt) in the municipality of João Pessoa, Paraíba (PB), northeastern Brazil. The primary care network is decentralized, demarcated by five Health Districts (HD), and the research was carried out in one of them.

### Population and Selection Criteria

The study was carried out with nurses who worked in the FHt of Health District I, which had 47 FHt.

The inclusion criteria for participation were to be a nurse working in the HD I FHt, and in the second phase, when the same instruments used before the educational intervention were applied, only nurses who took part in the proposed intervention with an attendance of at least 75% remained in the study. Four nurses who didn’t return to the other meetings were excluded, and 13 who didn’t attend the intervention were considered losses. A total of 30 nurses took part before and after the intervention.

### Data Collection

Data collection happened between November 2020 and March 2021, in two stages: before the educational intervention and one month after its completion. Initially, nurses were interviewed to find out what they needed to know about growth and development monitoring in childcare, in order to design the instrument and select the content to be covered in the intervention.

The data collection tool was built in accordance with the topics suggested in the interviews with nurses and with the guidelines for child health care, as well as the scientific literature on child care in childcare consultations in primary care. It is divided into two parts: the first contains data on the characterization of the participant, and the second part is divided into sections: to assess the nurses’ knowledge, with 18 questions, and to assess their practice, with 22 objective multiple-choice and discursive questions, containing the following dimensions of care: periodicity of consultations; feeding the child; physical examination; assessment and alteration of growth; assessment and alteration of neuropsychomotor development; health education; care for children who are victims of violence and care for children with special health needs.

This tool was assessed through content validation in a single round by 13 judges with experience in child health, in order to assess whether the items were consistent with the criteria of clarity, representativeness and relevance. After the instrument was returned by the judges, all the suggestions were analyzed and discussed among the researchers, and some items were reformulated and others excluded, according to the judges’ suggestions. The Content Validation Index was used with Bayes inference and a 95% confidence interval^(16)^. The instrument’s internal consistency was verified by the Cronbach’s alpha test, in which the knowledge section had a coefficient of 0.779 for the clarity criterion, and 0.973 for the practice section for the relevance and representativeness criterion.

Most of the items in the instrument obtained a Bayes Content Validation Index above 90% in the Clarity and Representativeness criteria. The overall Cronbach’s alpha for clarity in the knowledge section was 0.779 and for relevance and representativeness was 0.750; in the practice section it was 0.935 and 0.973, respectively.

### Intervention

The nurses were invited to participate in the intervention after an agreement was reached with the HD’s technical management, who authorized the release of these professionals.

The workshops took place in the HD meeting room, where the research and the intervention program were presented to the nurses. They were held with two groups concurrently, with three meetings in each group, in the afternoon, once a week, lasting three hours each and a remote complementary activity, to carry out an exercise, totaling 10 hours. The content covered in the workshops was: childcare consultations; monitoring child growth and development; as well as a brief explanation of child nutrition and violence, which was prepared using the relevant child health literature and the child health care guidelines as a reference.

Due to the Coronavirus Disease (COVID-19) pandemic, the workload of the planned intervention had to be reduced so that it could be carried out. The biosafety and prevention measures against the new coronavirus recommended by national and international authorities were adopted at all stages of data production.

At the first meeting, the data collection instrument was applied before the intervention and 18 nurses took part in the first group and 12 in the second, making a total of 30 nurses. Notebooks and handouts containing the main content covered in the intervention were given to the nurses so that they could consult them when necessary. The materials given to the nurses were the same for both groups.

The teaching method in the workshops was based on Ausubel’s Significant Learning Theory, which used the concepts of conceptual hierarchy as strategies for selecting what would be worked on in the intervention; the relevance of prior knowledge, which should be related to the new knowledge for significant learning to occur^(17)^; and prior organizers that serve as an anchor for new learning^(13)^.

The face-to-face theoretical activities were developed through dialogic exposition, with the use of slides and video presentations, exchange of experiences, and practical activities, using materials to assess the growth and development of children’s health. The nurses were encouraged to participate actively in the workshops, by promoting moments of group discussion, exposing their experiences and possible problems identified in the day-to-day running of the health service. It is important to highlight that meaningful learning requires the individual to have a supportive and active attitude, capable of attributing their own meanings to the content they assimilate, broadening their knowledge^(17)^.

At the last meeting, an overall evaluation of the workshop was carried out, when the nurses assessed the intervention and the meaning it had for them. A month after the workshops had been held, the second stage of data collection began, when the same instrument was applied individually in the health units in order to reassess nurses’ knowledge and practice of child growth and development monitoring.

### Data Analysis and Processing

The Statistical Package for the Social Sciences (SPSS), version 20, was used for statistical analysis. Descriptive analysis (relative and absolute frequencies, mean, median, standard deviation) and inferential analysis were done using the Wilcoxon proportion and non-parametric tests, as the variables had a normal distribution, and the difficulty indices for each item in the pre- and post-intervention situation, according to the Item Response Theory (IRT) with the Rasch model, which presents a difficulty index for each item before and after the intervention^(18)^.

It is noteworthy that some items did not show a significant difference, which can be explained by the fact that the test did not identify a difference, but that significance may exist, therefore the overall significance of the instrument provided by the Wilcoxon test is considered more important. Furthermore, it is the Rasch index that will define the difficulty of answering the item, because it measures the degree of difficulty that the nurse felt in the test. When it is lower, it means that the intervention favored learning, as the nurses found it easier to answer the items in the post-test.

It is worth noting that when it was not possible to calculate the Rasch index (difficulty index) for the item, a comparison was made with the number of correct answers. In all statistical hypotheses, a significance level of 0.05 was considered.

### Ethical Aspects

The participants agreed to take part in the study by signing the Informed Consent Form (ICF). The study was cleared by the Research Ethics Committee under Certificate of Submission for Ethical Appraisal 3773616, in accordance with the requirements established by Resolution 466/2012 of the National Health Council, and all participants signed the ICF.

## RESULTS

In a first step we present the data characterization of the study participants, for which no correlations were established with the intervention data, but it is important to give an idea of their profile. Of the 30 nurses taking part, only two were male, ten were aged between 31 and 40 (33.3%), 13 (43.3%) had more than 20 years’ training and 17 (56.7%) had been working in the ESF for more than ten years. In terms of employment, 23 (73.7%) were not permanent employees, only eight nurses were permanent employees and 21 (70%) had specializations.

In the pre-intervention situational diagnosis, shown in Table 1, the nurses had limited knowledge, especially in the areas of physical examination and frequency of consultations. In addition, when observing the percentage of correct answers to the items in the knowledge section by the nurses, before and after the intervention, it should be noted that there was a significant difference in five items (p < 0.05). As for the difficulty indices of the Rasch IRT model, most of them showed lower values after the intervention.

**Table 1:** Number of correct answers by nurses (n = 30) for items belonging to the knowledge section and the difficulty indices before and after the educational intervention – João Pessoa, PB, Brazil, 2020.

Items^‡^	Pre test		Post test		p-value*	IRT^†^	
	n	%	n	%		Pre test	Post test
What is the ideal time for a home visit when a healthy newborn is discharged from the maternity hospital?	4	13,3	22	73,3	**<0,001**	2,07	–1,14
What is the minimum number of childcare visits recommended in the first year of life?	9	30,0	19	63,3	**0,031**	0,95	–0,63
The Ministry of Health recommends that appointments be made periodically and also according to the child’s needs. Choose the correct alternatives.	3	10,0	19	63,3	**<0,001**	2,42	–0,63
When assessing infant feeding, which of the following indirect indications can alert you to the fact that the child is not receiving enough breast milk.	13	43,3	21	70,0	0,115	0,30	–0,96
In relation to the introduction of food to children from the age of six months, analyze the following statements.	10	63,3	24	80,0	0,227	–0,62	–1,55
When should a complete physical examination of the child be carried out at the childcare appointment?	1	3,3	19	63,3	**<0,001**	3,63	–0,63
How should the physical examination of the child be carried out at the routine appointment?	4	13,3	18	60,0	**0,001**	2,07	–0,47
In relation to child growth monitoring, check T (True) and F (False).	11	36,7	10	33,3	0,999	0,61	–0,96
In the case of infants under one year of age with a low weight-for-age situation (below Score-2 on the chart), identified for the first time at the childcare visit, which of the behaviors would be appropriate.	18	60,0	21	70,0	0,581	–0,46	–1,14
The monitoring of preterm infants (<37 weeks gestational age) requires.	17	56,7	24	80,0	0,092	–0,3	–3,61
In relation to the monitoring of child development, mark T for True and F for False.	23	73,7	22	73,3	0,999	–1,33	–1,34
Among the risk factors listed, which one is not associated with neuropsychomotor developmental deficit (NPMD) in children?	28	93,3	29	96,7	0,999	–2,87	–3,62
From what age group is a child expected to sit without support?	23	73,7	23	76,7	0,999	–1,33	–2,87
How important is the mother’s/caregiver’s opinion in monitoring the child’s development?	29	96,7	29	96,7	0,999	–3,62	–2,07
What should you do at a childcare appointment when a child is developing adequately but has risk factors for developmental changes?	11	36,7	13	43,3	0,791	0,61	–2,87
In relation to the warning signs for children under 2 months of age, mark V for True and F for False.	26	86,7	28	93,3	0,625	–2,07	–1,14
What is the appropriate course of action when caring for a child in a situation compatible with violence?	25	83,3	26	86,7	0,999	–1,79	–0,63
How should continuity of care for children with special health needs (chronic illness, autism, hyperactivity, premature babies, etc.) be handled in the FHS?	23	76,7	28	93,3	0,125	–1,33	–0,63

*p-value = significance level of the proportion test; †IRT = Item Response Theory; ‡The continuity of the questions can be consulted in supplementary material.

Regarding the pre-intervention situational diagnosis of the nurses’ practice, Table 2 shows gaps, especially in the questions relating to the dimensions of growth assessment and health education. In addition, comparisons are shown of the correct answers to the items answered in the practice section by the nurses, before and after the intervention. Only the item “Name at least three danger signs” showed a statistically significant difference (p < 0.05). In relation to the difficulty indices in the post-intervention, all were lower than in the pre-intervention, with the exception of the item “Do you routinely monitor child development at childcare appointments?”, which also had a lower percentage of correct answers in the post-intervention. The difficulty index in the post-test of the IRT for some items in the practice section could not be analyzed, as all the nurses got the answers right in the post-intervention, not allowing for comparisons.

**Table 2:** Number of correct answers by nurses (n = 30) and the difficulty indices of the items in the pre- and post-intervention practice section - João Pessoa, PB, Brazil, 2020.

Items^‡^	Pre test		Post test		p-value*	IRT^†^	
	n	%	N	%		Pre test	Post test
How often do you visit the newborn at home?	8	26,7	11	36,7	0,503	1,14	0,63
How often do you visit children under 1 year of age?	26	86,7	27	90,0	0,999	–2,08	–2,48
How often do you visit children between the 2nd and 3rd year of age?	21	70,0	22	73,3	0,999	–0,96	–1,17
How do you assess the child’s diet?	25	83,3	27	90,0	0,687	–1,80	–2,48
How do you advise mothers/caregivers about the child’s diet?	28	93,3	29	96,7	0,999	–2,89	–3,70
Do you carry out a physical examination of the child at the first childcare appointment?	28	93,3	30	100	0,472	–2,89	–
What anthropometric measurements do you usually consider when assessing a child’s growth?	18	60,0	25	83,3	0,065	–0,46	–1,84
What do you do when you identify a change in the child’s weight?	20	66,7	26	86,7	0,774	–0,79	–1,18
Do you routinely monitor child development at childcare appointments?	27	90,0	26	86,7	0,999	–2,43	–2,13
Which item do you use the most to assess the child’s development?	27	90,0	30	100	0,236	–2,43	–
What is your main justification for asking mothers/caregivers what they think of their child’s development?	24	80,0	28	93,3	0,219	–1,56	–2,94
How do you assess the child’s development during the childcare visit?	24	80,0	27	90,0	0,453	–1,56	–2,48
Do you advise mothers/caregivers on how to stimulate the child’s development?	27	90,0	30	100	0,236	–2,43	–
Where do you record the child’s growth and development data?	26	86,7	30	100	0,120	–2,08	–
Do you give guidance to mothers/caregivers on the results of the child’s growth assessment?	26	86,7	30	100	0,120	–2,08	–
Name two guidelines for assessing growth.	16	53,3	23	76,7	0,092	–0,15	–1,38
Do you know the danger signs / warning signs of a sick child?	27	90,0	30	100	0,236	–2,43	–
Name at least three danger signs.	14	44,7	25	83,3	**0,013**	–0,15	–1,84
Do you ever talk to mothers/caregivers about accident prevention?	27	90,0	29	96,7	0,325	–2,43	–3,70

*p-value = significance level of the proportion test; ^†^IRT = Item Response Theory; ^‡^The continuity of the questions can be consulted in the supplementary material.

When comparing the nurses’ knowledge and practice scores before and after the intervention (Table 3), the data showed a significant difference in the nurses’ scores on all the items, i.e. an increase in the scores expressed through the descriptive measures: mean and median. The IRT with the Rasch model also showed that there was a significant reduction in the difficulty index when taking the test after the intervention.

**Table 3:** Comparison between the correct answers in the knowledge and practice section for nurses (n = 30) and the difficulty in answering the items before and after the educational intervention – João Pessoa, PB, Brazil, 2020.

Variable	Intervention	Mean	SD	Median	p-value
Nurses’ knowledge	Pre	9,57	1,99	9,50	<0,001*
	Post	13,17	2,07	13,0	
Degree of difficulty of the knowledge section	Pre	–0,17	1,93	–0,38	<0,001^†^
	Post	–1,49	1,05	–1,14	
Nurses’ practice	Pre	76,92	18,70	86,7	
	Post	86,70	15,63	86,7	0,002*
Degree of difficulty of the practice section	Pre	–1,60	1,14	–2,08	
	Post	–2,05	1,16	–2,13	0,002^†^

*Wilcoxon test significance level; ^†^IRT = Item Response Theory.

## DISCUSSION

The National Policy for Comprehensive Child Health Care is the leading early childhood policy, and one of its principles is comprehensive care. Comprehensive care for children includes all promotion, prevention, treatment, rehabilitation and care actions, and also considers access to all levels of care, with monitoring of the child’s entire itinerary in a network of care and social protection^(19)^.

The knowledge and practices of nurses working in PHC, in the context of the HFts, with regard to growth monitoring consist of measuring weight, length, head circumference, measuring BMI and recording growth curves in the Child Handbook (CH). For developmental surveillance, the child’s motor, communication, social interaction and cognitive skills should be assessed, according to the milestones contained in the CHD, and risk factors, physical examination, clinical history and family context should also be taken into account; in addition to assessing vaccinations and supplements, and health education practices^(3,19)^.

The proposed educational intervention on surveillance of growth and development during childcare appointments was effective because, although the number of correct answers remained low for some of the items, there was an increase in the number of correct answers and a decrease in the difficulty index, showing that nurses found it easier to answer the items after the intervention.

In this study, the initial assessment showed that there were gaps in the nurses’ performance during childcare appointments, especially in relation to their knowledge on the subjects of the periodicity of the appointment and physical examination, and the practice of health education, indicating the need for training in line with what is expected for this practice.

Nurses’ knowledge of the periodicity of appointments recommended by the Ministry of Health is key. However, when transferring the domain of knowledge to the domain of practice, the number of correct answers in this last section, after the intervention, is still low, especially with regard to the first appointment, which suggests that nurses are still not following the ideal time for appointments recommended by the Ministry of Health, even though they know what the recommendation is.

This is an aspect that deserves to be reinforced among nurses, as evidence shows that children who attend routine follow-up appointments on a regular basis are twice less likely to have developmental complaints as those who attend unscheduled appointments^(20)^.

A study carried out in the Brazilian city of Rio Branco, in the state of Acre, found that the number of annual appointments corresponded to only 6.5% of what is recommended, which may be reflected in the low prevalence of immunizations and exclusive breastfeeding. In addition, this population had a high mortality rate due to malnutrition and pneumonia, and a high number of hospitalizations due to respiratory infections in childhood^(21)^. This shows the importance of children’s attendance at childcare appointments, as this is a powerful tool for promoting health and preventing childhood illnesses.

As an investigative process, the childcare nursing visit makes it possible to get to know the child and their family, as well as to assess the child through physical examination, using the propaedeutic method in a systematic way to promote comprehensive care^(6)^. However, a study shows weaknesses in the care provided by nurses in childcare appointments, since the absence of a physical examination reveals weaknesses in the appointment. As a result, the child’s state of health will not be fully assessed, as recommended by the child health care guidelines^(8)^.

Thus, it is understood that the better knowledge demonstrated in the study about the physical examination can be reflected in the better care offered to children, because educational interventions lead to an increase in knowledge and reflect changes in practice, especially when they focus on the skills required by the participants^(11)^, as in the case of the educational strategy carried out.

It is understood that using SLT in the workshops with nurses favored the recovery and transformation of knowledge about aspects involving the surveillance of growth and development, since meaningful learning is a teaching-learning process that occurs when new knowledge relates and interacts with specific pre-existing knowledge, present in the individual’s cognitive structure, acquiring a new meaning and modifying pre-existing concepts continuously, in a non-arbitrary and non-literal (substantive) way^(17)^.

With regard to nurses’ practice in childcare consultations, this was assessed using an instrument with items related to the actions carried out in childcare appointments, which showed an increase in the number of correct answers. Thus, as the nurses already had prior knowledge about monitoring child growth and development and were interested in acquiring new knowledge and changing their daily practice, this had an influence on the results, which were favorable. Consequently, learning was significant, based on the principle that this occurs when the individual actively decides to expand their knowledge, based on what is significant to them, corroborated by the positive figures after the intervention. Furthermore, when teaching is based on the student’s existing knowledge, this is an important factor that will serve as an anchor for new concepts and, consequently, learning will take place^(13)^.

In the practice section, the items relating to “danger signs”; “what anthropometric measurements do you usually consider when assessing the child’s growth?”; and “guidance on the assessment of growth provided” were the items that showed the greatest increase in correct answers in the post-intervention period. A similar aspect was found in a study carried out in the Netherlands, in which training nurses in preventive health care for children resulted in a specific improvement in the skills needed to carry out these tasks^(22)^.

Most of the items relating to neuropsychomotor development in the knowledge and practice section had a high number of correct answers among the nurses. This is in contrast to a study which identified nurses’ lack of knowledge in carrying out developmental assessments, as well as their misunderstanding of the term neuropsychomotor development, referring to the child’s general condition and growth measurements, rather than the assessment of developmental milestones for age and the risks of delay^(23)^.

It should be remarked that, although the professionals in this study demonstrated knowledge and good practices regarding the relevance of caregivers’ opinions on children’s development, a survey showed that 61.1% of the mothers or guardians of the children in the sample reported never being asked about their perceptions of children’s growth and development^(24)^.

A study carried out in the United States revealed that, based on reports from parents of children on the autism spectrum, before their diagnosis they felt that their concerns about their children’s development were not taken into account by the professionals who monitored their growth and development^(25)^.

Thus, it is hoped that the results presented will provide a new reality regarding the assessment of child growth and development by nurses in childcare consultations and that the lesser difficulty shown in answering the items after the training will reflect the professionals’ awareness of the importance of including parents and caregivers in the monitoring of child development, favoring dialogue and comprehensive care.

It is worth noting that some of the items in the tool had a high number of correct answers before the intervention, which remained high afterwards, both because the questions were easy to answer and because they are common themes in nurses’ practice. On the other hand, the promising results of the educational intervention are also noticeable, considering the statistically significant difference in the number of correct answers in the knowledge and practice sections, as well as the significant decrease in the difficulty index in answering the item, which shows that nurses had less difficulty answering the instrument after the workshop.

Thus, it is understood that training has the potential to qualify health professionals, especially nurses, to provide more comprehensive care for children, while at the same time improving care practices^(26)^ and keeping professionals up to date.

However, the nurses taking part in the intervention show that they have not taken part in continuing education programs focused on children’s health, which requires support, investment and funding from the organizations responsible for health services, which must make continuing education accessible to nurses in order to offer quality nursing care^(27)^.

Further research is therefore needed, as the willingness of professionals to learn and the provision of training are perhaps the biggest gaps identified in this study. Therefore, spaces for reflection need to occur routinely in care practice and not just at specific moments, and they need to be extended to all members of the FHS team, where many children in vulnerable situations are cared for. Other challenges for nurses in this group are the lack of necessary supplies, adequate physical structure and time to carry out childcare as recommended due to the high demand from the FHS, which could be important factors for expanding the research.

It is clear that applying the SLT in health courses is a strategy to recover the previous knowledge of health professionals in their work environments, as well as to qualify them in order to seek to expand and disseminate knowledge in a way that is closer to reality, and provide transformation in practice and quality of care.

For this purpose, it is essential to consider students as biopsychosocial beings and enable them to actively participate in the teaching-learning process, to make the knowledge meaningful in their cognitive structure and transfer it to the various situations in their life context^(28)^.

The study’s limitations are related to the length of the intervention, as well as the fact that the research was carried out with a small group, in which the group itself was the control, so there is no equivalence for the changes observed in the responses to be attributed solely to the effect of the intervention, so it is not possible to generalize the findings and establish the causality of the intervention. However, the findings of this study were similar to those that used a control group or not, which showed successful effects on professionals’ knowledge and practices^(29,30)^. In addition, some nurses may have been afraid to answer their real situation in the practice section of the instrument, thus making it impossible to draw greater inferences about their care reality.

The study contributes to the training process in health and nursing by highlighting the importance of ongoing health education on the monitoring of growth and development from the perspective of integrality carried out in childcare in Primary Health Care, the gateway to health services, avoiding the aggravation of various diseases prevalent in childhood and their hospitalization for conditions sensitive to primary care.

## CONCLUSION

The study showed that the educational intervention for the knowledge and practice of nurses who work in PHC, for the surveillance of growth and development in the childcare visit, based on the SLT, had positive effects, which was noticeable in the increase in the scores of correct answers to the items, which became possible to be more comprehensive in the child’s follow-up actions and in the reduction in the difficulty index in answering the items in the knowledge and practice section after the workshops were held, confirmed by the association of the statistical tests before and after the intervention. This was confirmed by the association of the statistical tests before and after the intervention.

It is believed that the use of the SLT helped to conduct the workshops, as well as helping to understand the importance of considering the professionals’ prior knowledge, stimulating discussions to facilitate learning, in line with the nurses’ reality. However, it is up to the professionals to decide to expand their knowledge and improve their practice based on what is significant to them, something we hope has indeed happened.

It is expected that the study will sensitize managers to carry out training on this subject and to encourage continuing health education in order to improve the care offered to children in Primary Care services, where many vulnerable children receive their care.
